# Disulfidptosis contributes to rotenone-induced dopaminergic neuron damage

**DOI:** 10.4103/NRR.NRR-D-25-00024

**Published:** 2025-10-30

**Authors:** Wenqi Ye, Qifu Zhang, Wei Ge, Haoyin Liu, Yaohui Shan, Feng Ye, Xiaogang Wang, Yuanpeng Zhao, Guorong Dan, Mingliang Chen, Yan Sai

**Affiliations:** Institute of Toxicology, College of Preventive Medicine, Third Military Medical University, Chongqing, China

**Keywords:** cell death, cystine, cytoskeleton, disulfidptosis, dopaminergic neuron, neurodegeneration, oxidative stress, Parkinson’s disease, rotenone, SLC7A11

## Abstract

Parkinson’s disease is a neurodegenerative disorder whose pathogenesis remains incompletely understood. Rotenone exposure is reportedly associated with Parkinson’s disease. In addition, disulfidptosis is a newly identified form of cell death. Interestingly, an analysis of the Gene Expression Omnibus Parkinson’s disease database indicated that approximately 30 genes that are significantly altered in patients with Parkinson’s disease are associated with disulfidptosis. In the present study, using proteomics, a number of important proteins related to disulfidptosis were identified as significantly altered in rotenone-exposed dopaminergic neurons. Further analysis revealed that the formation of abnormal disulfide bonds was also increased in rotenone-exposed dopaminergic neurons. The protein expression of solute carrier family 7 member 11 and amino acid transporter heavy chain SLC3A2 was upregulated in rotenone-exposed dopaminergic neurons, and was correlated with extracellular matrix protein 1 protein expression. These findings indicate that in rotenone-exposed PC12 cells, a cystine influx is triggered, and the conversion of cystine to cysteine is inhibited by a reduction in the oxidized nicotinamide adenine dinucleotide phosphate/reduced nicotinamide adenine dinucleotide phosphate ratio, which leads to cystine accumulation. This excessive accumulation of cystine then promotes the formation of abnormal disulfide bonds in cells, ultimately resulting in disulfidptosis of rotenone-exposed dopaminergic neurons. In this process, the Ras-related C3 botulinum toxin substrate 1/WAVE regulatory complex/actin-related protein 2/3 pathway was markedly activated, which led to the collapse of the cytoskeleton in rotenone-exposed PC12 cells. Together, our findings suggest that rotenone may induce solute carrier family 7 member 11 expression through extracellular matrix protein 1 activation to cause cystine accumulation, which results in disulfidptosis characterized by cytoskeleton collapse. The present results provide new perspectives for research into neurodegenerative diseases.

## Graphical abstract

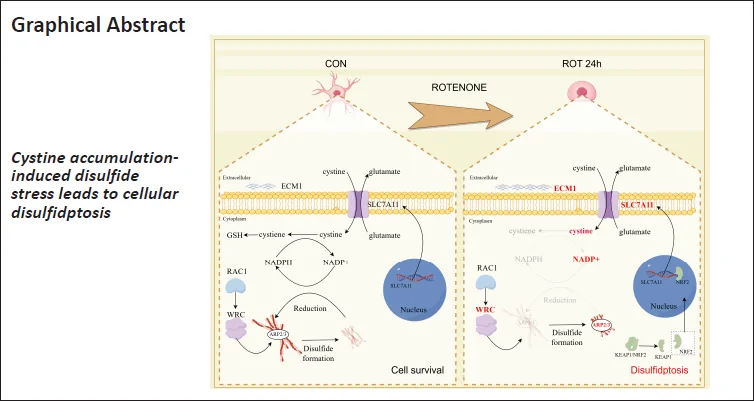

## Introduction

Parkinson’s disease (PD) is a common neurodegenerative disorder in middle-aged and older individuals; it mainly manifests as motor symptoms such as slow movement, tremor, muscle stiffness, and abnormal gait (Tanner et al., 2011; Vallerga et al., 2020; Stern et al., 2022; Wang et al., 2024). The incidence of PD is also increasing year by year, and presents a substantial challenge for patients and their families. It is currently thought that neuroinflammation, apoptosis, oxidative stress, cellular stress response, endoplasmic reticulum stress, neurotoxicity, and autophagy may be involved in the main mechanism of dopaminergic neuron degeneration and damage in PD (Seppi et al., 2019; Sen et al., 2022; Roy et al., 2023; Saleh et al., 2024). Mitochondrial dysfunction plays an especially important role in PD pathogenesis.

Rotenone is a natural compound that is a mitochondrial complex I inhibitor. It can inhibit mitochondrial respiration and trigger mitochondrial dysfunction (Chakrabarti et al., 2022; Huang et al., 2022; Xiao et al., 2023; Wang et al., 2024; Yamamoto et al., 2024). Long-term rotenone exposure is reportedly associated with PD (Tanner et al., 2011; Saleh et al., 2024). Moreover, numerous studies have successfully constructed animal models of PD using rotenone. Animals in these models exhibit symptoms similar to those of PD, and dopaminergic neurons undergo degenerative death in the substantia nigra of the midbrain (Mello et al., 2022; Roy et al., 2023; Linjacki et al., 2024; Ranasinghe et al., 2024). To date, however, the mechanism of rotenone-induced PD-like disease remains unclear.

In addition to well-characterized forms of regulated cell death, such as apoptosis, necroptosis, ferroptosis, and pyroptosis, recent studies have identified disulfidptosis as a novel cell death mode that is driven by dysregulated disulfide bond formation and cytoskeletal protein aggregation (Liu et al., 2023). Unlike apoptosis, which involves caspase activation and DNA fragmentation, disulfidptosis is characterized by disulfide stress and cytoskeletal collapse without caspase dependency. In addition, necroptosis relies on receptor-interacting serine/threonine-protein kinase (RIPK)1/RIPK3/mixed lineage kinase domain like pseudokinase signaling, and pyroptosis involves gasdermin-mediated membrane pore formation. By contrast, disulfidptosis operates via distinct molecular mechanisms (Mao et al., 2024). Furthermore, although both ferroptosis and disulfidptosis involve redox imbalances, the latter is specifically triggered by aberrant disulfide bond formation rather than lipid peroxidation (Mao et al., 2024). These distinctions underscore the unique nature of disulfidptosis and its potential implications for neurodegenerative diseases, including PD.

Previous research has indicated that classic forms of cell death, such as apoptosis and necrosis, are important in rotenone-induced dopaminergic neuron degeneration and damage (Li et al., 2003; Mello et al., 2022; Linjacki et al., 2024; Ranasinghe et al., 2024). In recent years, a new type of cell death, known as disulfidptosis, has been identified. Disulfidptosis is a form of cell death that is triggered by specific metabolic stress and alterations in redox status (Liu et al., 2023; Ma et al., 2023; Mao et al., 2024). Disruption of the intracellular disulfide bond metabolism results in protein misfolding and aggregation, and cytotoxic reactions are subsequently triggered: this process is termed disulfidptosis (Hu et al., 2023; Song et al., 2025). As a novel form of cell death, disulfidptosis is distinct from traditional apoptosis, necrosis, and other forms, thus providing a new perspective for understanding cell fate regulation (Wang et al., 2023b; Zhong et al., 2023). To date, studies have demonstrated that disulfidptosis may play an important role in the occurrence and development of various diseases, including cancer and neurodegenerative diseases (Liu et al., 2023; Ma et al., 2023; Shuai et al., 2024; Song et al., 2025). However, research into the underlying molecular mechanisms and the biological importance of disulfidptosis remains in its early stages. Disulfidptosis is closely associated with disordered intracellular disulfide bond metabolism. Cystine, which is an amino acid that contains disulfide bonds, plays an important role in this process (Koppula et al., 2018; Liu et al., 2022, 2023; Shuai et al., 2024).

Under normal physiological conditions, the metabolism of cystine within cells is crucial for maintaining the redox balance (Vallerga et al., 2020; Zhong et al., 2023). Continuous cystine accumulation inside cells ultimately results in cellular disulfide stress (Liu et al., 2023), which promotes the progression of disulfidptosis (Liu et al., 2020, 2023; Romani et al., 2022). Disulfidptosis is an orderly form of cell death that is initiated under specific conditions or environments; the initiation and completion of this process require the participation of a series of key regulatory proteins. Solute carrier family 7 member 11 (*SLC7A11*) is reportedly a key gene for this death mode (Liu et al., 2020, 2023; Zhong et al., 2023). The *SLC7A11* protein, also known as xCT carrier protein, is a transmembrane protein that is located on the cell membrane. It combines with the amino acid transporter heavy chain SLC3A2 protein to form a complex and participates in the amino acid transport process, especially in the transport of glutamate and cysteine (Vallerga et al., 2020; Wang et al., 2023a; Shuai et al., 2024).

The pathogenesis of dopaminergic neuron damage has been reported in terms of apoptosis and autophagy (Xiao et al., 2023; Wang et al., 2024). However, it remains unclear whether disulfidptosis-mediated cell death occurs in rotenone-induced dopaminergic neuron degeneration. In addition, it not yet known whether disulfidptosis occurrence can lead to the collapse of the cytoskeletal network in rotenone-induced neurodegeneration. It is also speculated that cystine content regulation may be a promising therapeutic target for rotenone-induced dopaminergic neuron damage. The present study therefore focuses on exploring the mechanisms of disulfidptosis and its role in the collapse of the cytoskeletal network in rotenone-exposed PC12 cells. Moreover, in-depth research was conducted into related mechanisms; specifically, the extracellular matrix protein 1 (ECM1) regulation of SLC7A11 protein expression and the activation of the Ras-related C3 botulinum toxin substrate 1 (RAC1)/WAVE regulatory complex (WRC)/actin-related protein (ARP)2/3 pathway after cystine accumulation, which in turn induces the formation of abnormal disulfide bonds in the cytoskeleton.

## Methods

### Analysis of the Gene Expression Omnibus database

We downloaded PD-related datasets (GSE7621, GSE205450, and GSE187012) from the Gene Expression Omnibus (GEO) database (https://www.ncbi.nlm.nih.gov/geo/) and conducted the analysis using a bioinformatic website (https://bioinformatics.com.cn/).

### Cell culture

PC12 cells (a rat adrenal medulla pheochromocytoma cell line) can be used to establish cell models of various neurological diseases. For example, by treating PC12 cells with neurotoxins or inducing specific gene mutations, cellular models of PD can be obtained (Xiao et al., 2023). In the present study, PC12 cells were obtained from the Cell Bank of Shanghai Institutes for Biological Sciences, Chinese Academy of Sciences (RRID: CVCL_0481, CSTR no. 19375.09.3101RATTCR9). The PC12 cells were cultured in Dulbecco’s Modified Eagle Medium containing 10% fetal bovine serum (FBS; VivaCell, Shanghai, China) and 1% penicillin-streptomycin (Hyclone, Logan, UT, USA) at 37°C in an environment with 5% CO_2_. Throughout all experiments, cells were passaged when their confluence reached 70%–80%, and the number of passages was limited to 15 or fewer.

### Cell treatment

PC12 cells were treated with rotenone (Sigma, St. Louis, MO, USA) at various concentrations (0, 0.1, 0.5, 1.0, and 1.5 μM) for 24 hours. Alternatively, they were divided into groups and treated for 24 hours as follows: 1.0 μM rotenone + 2 mM 2-mercaptoethanol (2-ME; a disulfide bond reducing agent, dissolved in 55 mM Dulbecco’s phosphate-buffered saline [PBS]; Solarbio, Beijing, China), 1.0 μM rotenone + 500 μM 2-deoxy-D-glucose (2-DG; a glucose analog; MedChemExpress, Monmouth Junction, NJ, USA), 1.0 μM rotenone + 250 μM Tris(2-carboxyethyl) phosphine (TCEP; a disulfide bond reducing agent; MedChemExpress), 1.0 μM rotenone + 250 μM dithiothreitol (DTT; a disulfide bond reducing agent; MedChemExpress), 1.0 μM rotenone + 50 μM sulfasalazine (SSZ; an inhibitor of SLC7A11; MedChemExpress) or 1.0 μM rotenone + 30 μM CK-666 (an inhibitor of ARP2; MedChemExpress).

### Animal care and treatment

Male Sprague–Dawley rats (weighing 300–350 g, 8–9 weeks of age) were purchased from the Army Medical University (Chongqing, China; license No. SCXK (Yu) 20170002). All experiments were approved by the Animal Ethics Committee of the Third Military Medical University on 12 March 2019 (approval No. AMUWEC2019137). Only male rats were chosen because female rats have an estrous cycle; the use of only male rats therefore helps to reduce the effects of physiological cycle changes on the experimental results, making them more stable and reproducible. The animals were placed in a room at 23 ± 2°C and were provided with sufficient water and food. The rats were randomly divided into two groups (15 rats per group): the control group and the rotenone exposure group. The control group received subcutaneous injections of dimethyl sulfoxide (DMSO) in the back at an equal volume to the rotenone group for 30 days. The rotenone exposure group received subcutaneous injections of rotenone in the back (2 mg/kg per day for 3 consecutive days, then 1 mg/kg per day for 7 consecutive days, then 0.5 mg/kg per day for 20 consecutive days). For tissue sampling, anesthesia was administered via the intraperitoneal injection of 35 mg/kg pentobarbital sodium (Sigma). The whole brains were rapidly removed and prepared for assay. The brain was washed with PBS, and the corpus striatum was dissected on ice according to the coordinates indicated in the rat brain atlas (Paxinos and Watson, 1998). Blood was collected from the apex of the heart using a syringe.

### Observation of cells under a light microscope and image acquisition

PC12 cells were incubated for 24 hours at 37°C with 5% CO_2_ in DMEM containing 10% FBS (VivaCell), 1% penicillin-streptomycin (Hyclone), and either 0 or 1.0 μM rotenone; there were three treatment groups for each condition. Imaging and observations were performed using an inverted light microscope (Olympus, Tokyo, Japan).

### Cell viability assay

The Cell Counting Kit-8 (CCK-8) method was used to determine cell viability. PC12 cells were seeded in a 96-well culture plate at a density of 1 × 10^4^ cells per well and were incubated for 24 hours (three replicate wells per treatment). After treatment, 10 μL of CCK-8 (Dojindo Laboratories, Kumamoto, Japan) solution was added to each well and incubated at 37°C for 1.5 hours. The absorbance value at 450 nm was measured using a microplate reader (SpectraMax M2E, Molecular Devices, Sunnyvale, CA, USA).

### *ECM1* RNA interference

To explore the specific mechanism of ECM1 in disulfidptosis, the following four sequences (5′–3′) were synthesized by Genepharma (Suzhou, Chian) for the ECM1 small interfering RNA (siRNA) experiments: *Ecm1*-Rat-288: sense, GTUCACCACUCCGAAACUUTT, antisense, AAG UTU CGG AGU GGU GAA CTT; *Ecm1*-Rat-765: sense, ACU GGC UAC UCU CAC CUU ATT, antisense, UAA GGU GAG AGU AGC CAG TTT; *Ecm1*-Rat-396: sense, CAC UUU CCU AAA CCC UAA UTT, antisense, AUU AGG GUT TIA GGA AAG UGT T; *Ecm1*-Rat-1525: sense, AGC UGC CAU ACC CAG AAC ATT, antisense, UGU UCU GGG UAU GGC AGC UTT. The PC12 cells were transfected with ECM1 siRNA for at least 24 hours using Lipofectamine 3000 (Invitrogen, Carlsbad, CA, USA), following the manufacturer’s instructions. Protein levels were assessed using western blot analysis after 48 hours of transfection.

### Quantitative reverse transcription-polymerase chain reaction assays

Total RNA was extracted from PC12 cells using TRIzol reagent (Sangon Biotech, Shanghai, China). First-strand cDNA was then synthesized using Hifair® III 1^st^ Strand cDNA Synthesis SuperMix for quantitative reverse transcription-polymerase chain reaction (qRT-PCR) (Yeasen Biotechnology, Shanghai, China). qRT-PCR was subsequently performed using Hieff® qPCR SYBR Green Master Mix (Yeasen Biotechnology) on the Agilent Mx3000P qPCR system (Agilent Technologies, Santa Clara, CA, USA). qRT-PCR was performed to evaluate the expression of *SLC7A11* (primer (5′–3′): forward, CCC AAG TGG TTC AGA CGA TTG, reverse, GAG TCT TCT GGT ACA ACT TCT AGT) and *SLC3A2* (primer (5′–3′): forward, ACA GAG ATG GTG GCA CAA GG, reverse, CAG ACC AGC TAT GCC TCT CG) in PC12 cells. The average standard deviation of all samples studied was 0.10 cycles. The reaction protocol was as follows: 95°C pre-denaturation for 30 seconds, one cycle; 95°C denaturation for 15 seconds, 60°C annealing for 60 seconds, 40 cycles. All genes were normalized using β-actin (primer (5′–3′): forward, CGG GAT CCC CGC CCT AGG CAC CAG GGT, reverse, GGA ATT CGG CTG GGG TGT TGA AGG TCT CAA) as the internal reference gene, and the 2^–ΔΔCt^ method was used to analyze the results.

### Detection of the oxidized nicotinamide adenine dinucleotide phosphate/reduced nicotinamide adenine dinucleotide phosphate ratio

PC12 cells were seeded in a six-well plate (1 × 10^6^ cells/well). After treatment, the nicotinamide adenine dinucleotide phosphate (NADP^+^)/reduced nicotinamide adenine dinucleotide phosphate (NADPH) ratio was measured according to the manufacturer’s instructions (Cat# S0179 Beyotime, Shanghai, China). The absorbance was read at 450 nm using a microplate reader (SpectraMax M2E, Molecular Devices).

### Cystine-fluorescein isothiocyanate live cell probe

Cystine-fluorescein isothiocyanate (FITC) dye is a green fluorescent imaging probe for live cells that can measure cystine uptake at a single-cell level. PC12 cells were seeded in a 15-mm dish with a laser confocal glass bottom at a density of 1 × 10^5^ cells per dish and were incubated for 24 hours. After treatment, 1 mL of complete medium containing 5 μM cystine-FITC (Sigma) was added to each dish and incubated at 37°C for 30 minutes. The dishes were then washed twice with PBS before being observed under a laser confocal microscope (Stellaris 5, Leica, Wetzlar, Germany). The average fluorescence intensity of cystine-FITC was measured using ImageJ software (Version 1.5.3, National Institutes of Health, Bethesda, MD, USA).

### Determination of cystine concentration using high-performance liquid chromatography

High-performance liquid chromatography (HPLC) (Waters, Milford, MA, USA) was used to measure the intracellular cystine content. Rat striatal tissue and PC12 cells were lysed in radioimmunoprecipitation assay (RIPA) buffer (Beyotime, Cat# P0013B). To achieve normalization across various groups, the protein concentration of each group was adjusted as the normalization standard. Specifically, the cystine peak value obtained from HPLC was normalized based on the protein concentration. The same method was applied to both cell and tissue samples. Chromatographic conditions were as follows: chromatographic column, Xbridge Peptide BEH C18 column (dimensions: 250 mm × 4.6 mm, particle size: 5 µm; Waters); column temperature, 30°C. Mobile phase A consisted of 50 mM sodium acetate solution (Nuoershi, Chengdu, China) mixed with acetonitrile (Honeywell International Inc., Charlotte, NC, USA) at a ratio of 93:7; the sodium acetate solution was adjusted to pH 6.5 using glacial acetic acid (Chuandong Chemical, Chongqing, China). The mobile phase B was acetonitrile (Honeywell International Inc.).

### Fluorescent staining of F-actin

Before treatment, PC12 cells were seeded in a 15-mm dish with a laser confocal glass bottom at a density of 1 × 10^5^ cells per dish. After treatment, the dish was washed with PBS, and the cells were fixed with 4% paraformaldehyde at room temperature (20–24°C) for 15 minutes. The fixed cells were then permeabilized with 0. 3% Triton-X-100 (Beyotime) in PBS at room temperature for 8 minutes. Next, the permeabilized cells were incubated with 100 nM tetramethylrhodamine isothiocyanate phalloidin (Yeasen Biotechnology) at room temperature for 30 minutes. The cell nuclei were stained with an anti-fluorescence quencher containing 4′,6-diamidino-2-phenylindole (DAPI; Beyotime) in the dark at room temperature for 10 minutes, and were then observed under a laser confocal microscope (Stellaris 5, Leica).

### Fluorescent staining of tubulin

PC12 cells were washed twice with PBS before being fixed using a 3.7% formaldehyde solution (Beyotime) at room temperature for 20 minutes. Following fixation, the samples underwent three 5-minute washes with PBS supplemented with 0.1% Triton-X-100 (Beyotime). Tubulin-Tracker (Beyotime) was then diluted with PBS containing 0.1% Triton-X-100 at a 1:100 ratio. Subsequently, the prepared working solution of Tubulin-Tracker was carefully dropped onto the slides, and the slides were incubated in the dark at room temperature for **~**60 minutes. After incubation, the slides underwent four 5-minute washes with PBS containing 0.1% Triton-X-100 before being observed under a fluorescence microscope (Stellaris 5, Leica).

### Western blot analysis

Rat striatal tissue and PC12 cells were lysed in RIPA buffer (Beyotime, Cat# P0013B) and protein concentrations were measured using a bicinchoninic acid assay (Beyotime, Cat# P0010). Protein samples (40 μg/lane) were separated using sodium dodecyl sulfate polyacrylamide gel electrophoresis (Beyotime) and were electrophoretically transferred onto a polyvinylidene fluoride (Merck Millipore, Darmstadt, Germany) membrane. Next, they were blocked with 5% skim milk (Beyotime) at room temperature for 2 hours before being incubated with specific primary antibodies at 4°C overnight. Primary antibodies were as follows: rabbit anti-ECM1 (1:2000, Proteintech, Wuhan, China, Cat# 11521-1-AP, RRID: AB_2261964), rabbit anti-SLC7A11 (1:2000, ABclonal, Woburn, MA, USA, Cat# A13685, RRID: AB_2760546), rabbit anti-SLC3A2 (1:2000, Proteintech, Cat# 15193-1-AP, RRID: AB_2254909), rabbit anti-Kelch-like ECH-associated protein 1 (KEAP1; 1:2000, Proteintech, Cat# 10503-2-AP, RRID: AB_2132625), rabbit anti-nuclear factor erythroid 2-related factor 2 (NRF2; 1:2000, ABclonal, Cat# A0674, RRID: AB_2757326), rabbit anti-NCK-associated protein 1 (NCKAP1; 1:2000, ABclonal, Cat# A12229, RRID: AB_2759105), rabbit anti-ARP2 (1:2000, Proteintech, Cat# 10922-1-AP, RRID: AB_2221854), rabbit anti-ARP3 (1:2000, Proteintech, Cat# A4514, RRID: AB_2863284), rabbit anti-capping actin protein of muscle Z-line subunit beta (CAPZB; 1:2000, ABclonal, Cat# 25043-1-AP, RRID: AB_2879867), rabbit anti-β-actin (1:100 000, ABclonal, Cat# AC038, RRID: AB_2863784), and rabbit anti-histone 3 (H3; 1:10000, Proteintech, Cat# 17168-1-AP, RRID: AB_2716755). The secondary antibody horseradish peroxidase–conjugated goat anti-rabbit immunoglobulin (Ig)G (1:10 000, ABclonal, Cat# AS014, RRID: AB_2769854) was incubated at room temperature for 1.5 hours, and β-actin was used as the internal reference control. The immunoreaction was visualized using a chemiluminescent horseradish peroxidase substrate (Millipore Corporation, Billerica, MA, USA) and a chemiluminescent system (Bio-Rad, Hercules, CA, USA). Band density was measured using ImageJ software (Version 1.5.3).

### Immunofluorescence and image analysis

PC12 cells were fixed with 4% paraformaldehyde at room temperature for 30 minutes. After being washed three times with PBS, the PC12 cells were permeabilized in 0.3% Triton-X-100 for 15 minutes before being blocked with 3% goat serum (Beyotime, Cat# C0265) at room temperature for 1 hour. The cells were then incubated with rabbit anti-SLC7A11 antibody (1:100, Proteintech, Cat# 26864-1-AP, RRID: AB_2880661) and mouse anti-ECM1 antibody (1:200, LSBio, WA, USA, Cat# LS-C390060) overnight at 4°C. Next, the cells were incubated with FITC-goat anti-rabbit IgG (1:500, ABclonal, Cat# AS011, RRID: AB_2769476) or Cy3-goat anti-mouse IgG (1:500, ABclonal, Cat# AS008, RRID: AB_2769088) at room temperature for 1 hour. PC12 cells were treated using the same method as described above regarding cell treatment. The cells were then incubated with rabbit anti-NRF2 antibody (1:100, ABclonal, Cat# A0674, RRID: AB_2757326) overnight at 4°C before being incubated with FITC-goat anti-rabbit IgG (1:500, ABclonal, Cat# AS011, RRID: AB_2769476) at room temperature for 1 hour. The cell nuclei were stained with DAPI, and fluorescently stained cells were then analyzed using a laser confocal scanning microscope (Stellaris 5, Leica).

### Detection of glutathione levels

According to the manufacturer’s instructions (Beyotime, Cat# S0053), PC12 cells were lysed with lysis buffer, and the optical density (OD) value of glutathione (GSH) was measured at 412 nm using a microplate reader (SpectraMax M2E, Molecular Devices). The corresponding concentration values were calculated based on the standard curve with the OD value.

### Detection of glucose levels

According to the manufacturer’s instructions (Beyotime, Cat# S0201S), rat striatal tissue and PC12 cells were lysed using cell and tissue lysate (for glucose detection; Beyotime). The OD value of glucose was then measured at 630 nm using a microplate reader (SpectraMax M2E, Molecular Devices). The corresponding concentration values were calculated from these OD values based on the standard curve.

### Hematoxylin and eosin staining

After establishing a Parkinson’s disease-like model in rats via rotenone intoxication, the striatum of the rats was harvested to prepare paraffin sections. Paraffin sections of the rat striatum were sequentially immersed in Environmental Friendly Dewaxing Transparent Liquid I (Servicebio, Wuhan, China, Cat# G1128-1L) for 20 minutes, Environmental Friendly Dewaxing Transparent Liquid II for 20 minutes, anhydrous ethanol (SCRC, Shanghai, China, Cat# 100092683) I for 5 minutes, anhydrous ethanol II for 5 minutes, and 75% ethyl alcohol for 5 minutes. They were then rinsed with tap water. Frozen sections were removed from a –20°C freezer, restored to room temperature, fixed with tissue fixating solution (Servicebio, Cat# G1101) for 15 minutes, and rinsed with running water. The sections were then treated with high-definition constant staining pretreatment solution for 1 minute. Next, sections were placed into hematoxylin solution (Servicebio, Cat# G1076) for 3–5 minutes before being rinsed with tap water. The sections were then treated with hematoxylin differentiation solution, rinsed with tap water, treated with hematoxylin bluing solution, and rinsed with tap water. Finally, the sections were placed in 95% ethanol for 1 minute, eosin dye for 15 seconds, absolute ethanol I for 2 minutes, absolute ethanol II for 2 minutes, absolute ethanol III for 2 minutes, normal butanol I for 2 minutes, normal butanol II for 2 minutes, xylene (SCRC, Cat# 10023418) I for 2 minutes, and xylene II for 2 minutes. The nucleus was stained blue and the cytoplasm was stained red. The sections were then scanned by Servicebio, and image acquisition was performed using the CaseViewer system (Budapest, Hungary).

### Transmission electron microscopy

PC12 cells were incubated for 24 hours in DMEM containing 10% FBS (VivaCell), 1% penicillin-streptomycin (Hyclone), and either 0 μM or 1.0 μM rotenone at 37°C in an environment with 5% CO_2_. There were three groups for each condition. The samples were sent to Servicebio for further analysis.

### Data-independent acquisition proteomics

PC12 cells were incubated for 24 hours in DMEM containing 10% FBS (VivaCell), 1% penicillin-streptomycin (Hyclone), and either 0 μM or 1.0 μM rotenone at 37°C in an environment with 5% CO_2_. There were three groups for each condition. The samples were sent to Applied Protein Technology (Shanghai, China) for further analysis. The interactions among the involved proteins were analyzed using STRING software (https://cn.string-db.org/). Moreover, KEGG enrichment analysis was applied for the identification of key genes and pathways involved in rotenone-induced PC12 cell damage via https://bioinformatics.com.cn/.

### Disulfide bond proteomics

PC12 cells were incubated in complete medium containing either 0 μM or 1.0 μM rotenone for 24 hours. There were three groups for each condition. Subsequently, the samples were sent to Bio-Tech Pack Technology Co., Ltd. (Beijing, China) for disulfide bond omics analysis.

### Statistical analysis

All presented data represent at least three independent experiments. Statistical analysis was conducted using IBM SPSS Statistics, version 20.0 (IBM Corp., Armonk, NY, USA) and GraphPad Prism, version 8.0 (GraphPad Software, Boston, MA, USA, www.graphpad.com). All results are expressed as the mean ± standard error of the mean. For comparisons between two groups, an unpaired Student’s *t*-test was used. For comparisons among multiple groups, one-way analysis of variance was applied followed by Tukey’s *post hoc* test for pairwise comparisons. *P* < 0.05 was considered significant.

## Results

### Rotenone increases disulfidptosis-related gene expression, disrupts the cytoskeleton, and induces the death of PC12 cells

**Figure 1:**
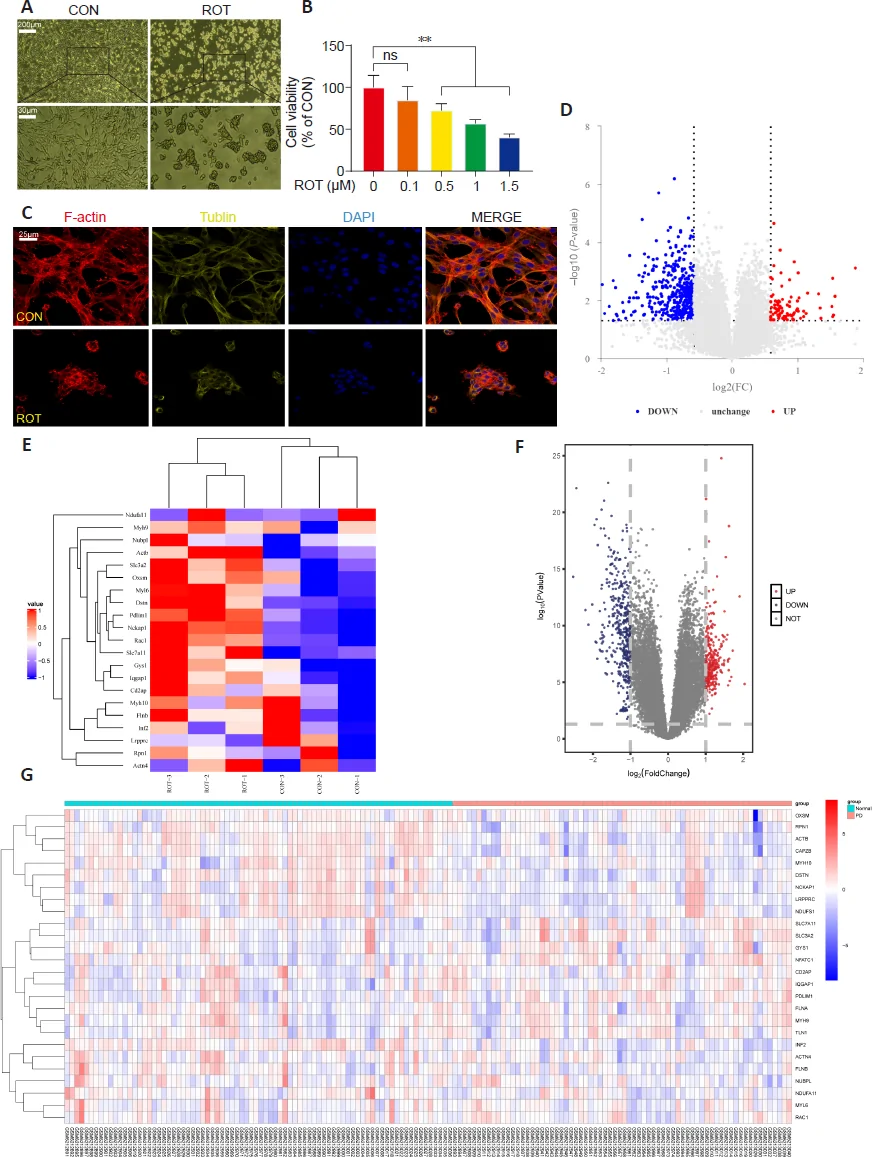
Rotenone exposure (ROT) increases the expression of disulfidptosis-related genes, disrupted the cytoskeleton, and induced the death of PC12 cells. (A) Morphology of control (CON) and ROT PC12 cells. (B) Viability of PC12 cells induced by different concentrations of ROT. (C) Cytoskeleton of CON and ROT PC12 cells; F-actin (red), tubulin (yellow), and 4′,6-diamidino-2-phenylindole (DAPI; blue). (D) Volcano plot of the proteomic analysis of ROT PC12 cells. (E) Heatmap of the expression of disulfidptosis-related proteins in ROT PC12 cells, analyzed using proteomics. (F) Volcano plot of the proteomic analysis of the Gene Expression Omnibus (GEO) Parkinson’s disease (PD) database. (G) Differences in disulfidptosis-related proteins in the human substantia nigra were analyzed using the GEO PD database. Values are presented as the mean ± standard error of the mean (*n* ≥ 3). **P < 0.01.

The morphology of PC12 cells transformed from a long fusiform shape into a quasi-circular shape in response to rotenone exposure (**Figure 1A**). Moreover, cell viability gradually declined with increasing rotenone concentrations (**Figure 1B**). The cytoskeleton of PC12 cells was observed to be severely damaged in rotenone-exposed PC12 cells (**Figure 1C**) compared with the control group. Proteomic analysis revealed that many disulfidptosis-related proteins were enriched (**Figure 1D** and **E**) in rotenone-exposed PC12 cells. Additionally, there were significant differences in many disulfidptosis-related genes when differential gene analysis was performed using PD-related data from the GEO database. Of these, differences were particularly prominent in SLC7A11 and its partner protein SLC3A2, which are the key regulator proteins in disulfidptosis (**Figure 1F** and **G**).

### Cystine accumulation is increased in rotenone-exposed PC12 cells

**Figure 2:**
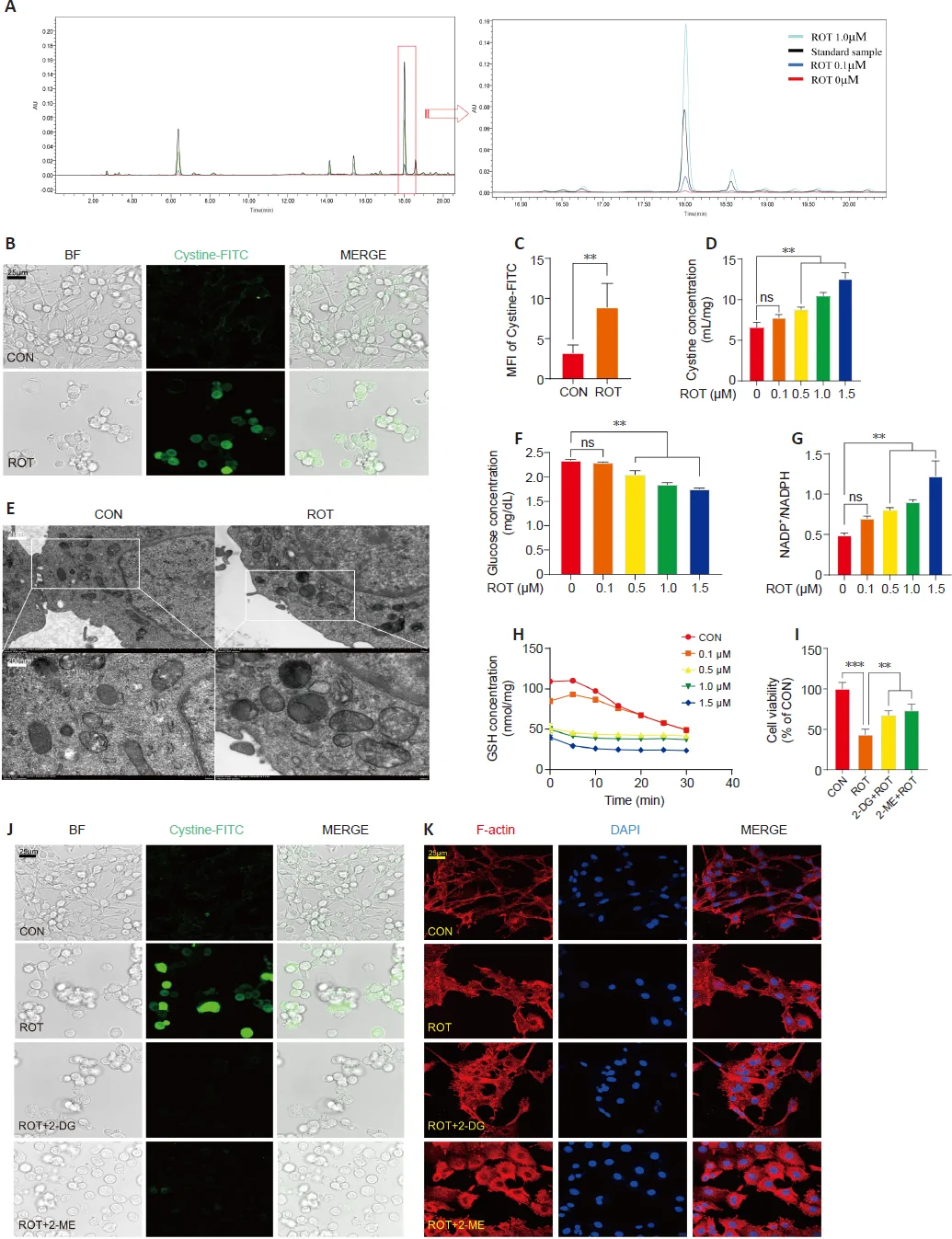
Cystine accumulation is significantly increased within rotenone-exposed (ROT) PC12 cells. (A) Peak diagrams of cystine content, detected using high-performance liquid chromatography, in PC12 cells exposed to different concentrations of ROT. (B) Cystine fluorescence was observed in live cells stained with cystine-fluorescein isothiocyanate (FITC, green) in control (CON) and ROT PC12 cells. (C) Average fluorescence intensity of cystine-FITC was calculated. (D) Using high-performance liquid chromatography, cystine content was detected in PC12 cells exposed to different concentrations of ROT. (E) Transmission electron microscope images of CON and ROT PC12 cells. (F) Glucose content in PC12 cells exposed to different concentrations of ROT. G Ratio of oxidized nicotinamide adenine dinucleotide phosphate (NADP^+^)/reduced nicotinamide adenine dinucleotide phosphate (NADPH) in PC12 cells exposed to different concentrations of ROT. (H) Glutathione (GSH) content in PC12 cells exposed to different concentrations of ROT for different times. (I) Cell viability of ROT PC12 cells treated with 2-deoxy-D-glucose (2-DG) or 2-mercaptoethanol (2-ME). (J) Cystine fluorescence was observed in live cells stained with cystine-FITC (green) in ROT PC12 cells treated with 2-DG or 2-ME. (K) Cytoskeleton of ROT PC12 cells treated with 2-DG or 2-ME. Values are presented as the mean ± standard error of the mean (*n* ≥ 3). **P < 0.01, ***P < 0.001. BF: Bright–field; GSH: glutathione; ns: not significant.

The intracellular cystine content increased significantly with increasing rotenone concentrations (**Figure 2A–D**). Furthermore, transmission electron microscopic observations revealed that the lysosomes appeared more swollen in rotenone-exposed PC12 cells than in control group cells, and the number of lysosomes was also increased with rotenone exposure (**Figure 2E**). Previous studies have reported that lysosomes are the main storage sites of cystine within cells (Adelmann et al., 2020). Additionally, the glucose content of rotenone-exposed PC12 cells gradually decreased with increasing rotenone concentrations (**Figure 2F**). The intracellular NADP^+^/NADPH ratio also increased with increasing rotenone concentrations (**Figure 2G**). However, the reduced GSH content was decreased (**Figure 2H**) in rotenone-exposed PC12 cells. In further experiments, the cell viability of PC12 cells was significantly reversed (**Figure 2I**) in rotenone-exposed PC12 cells treated with 2-DG or 2-ME. Moreover, the cystine content was significantly decreased (**Figure 2J**) and the cytoskeleton integrity was restored to some extent (**Figure 2K**) in rotenone-exposed PC12 cells treated with 2-DG or 2-ME. These findings suggest that rotenone exposure might lead to the accumulation of a large amount of cystine in PC12 cells, which might be cytotoxic. Furthermore, cystine accumulation may be associated with glucose metabolism.

### Solute carrier family 7 member 11 mediates the increased cystine uptake in rotenone-exposed PC12 cells

**Figure 3:**
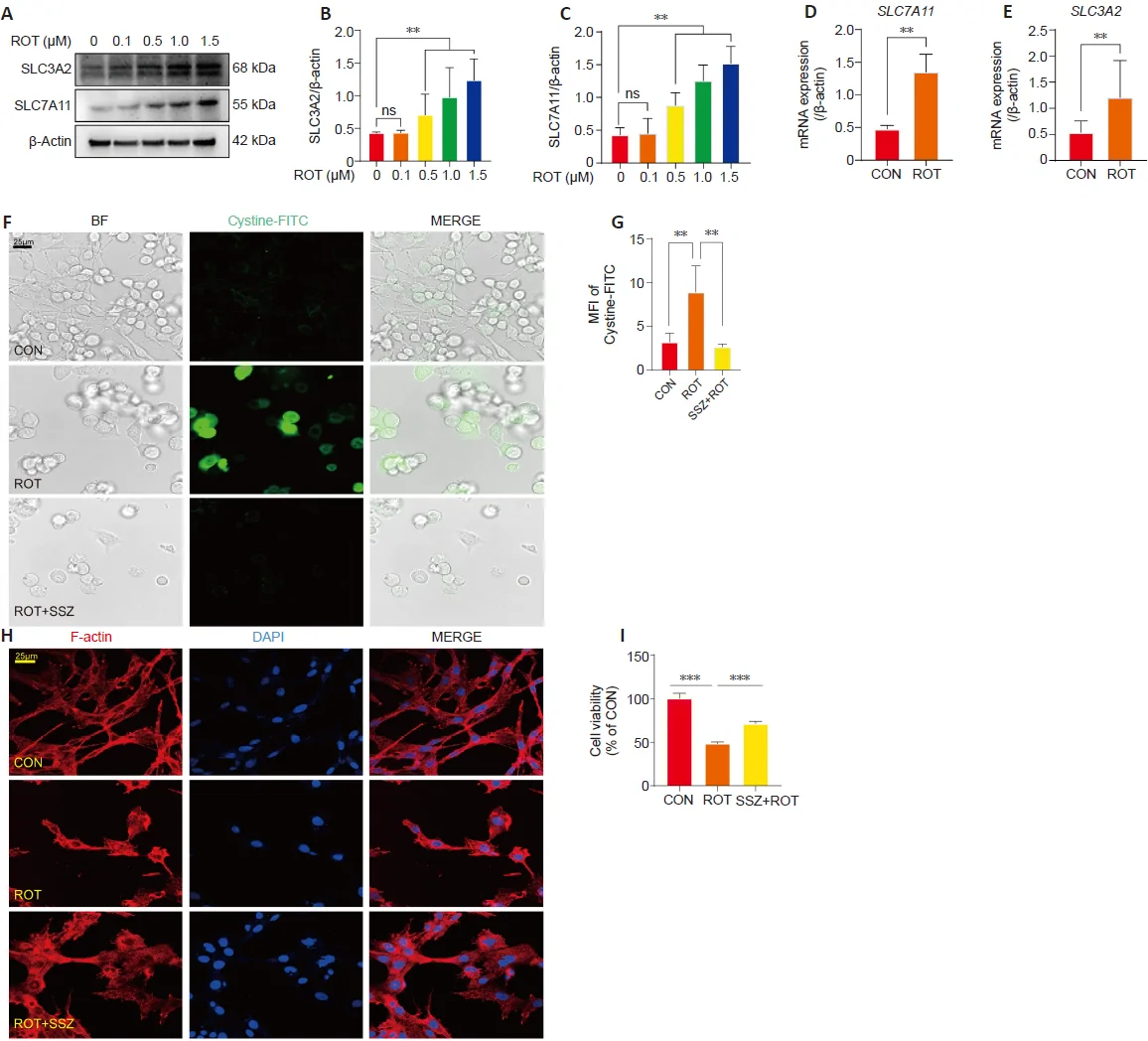
Solute carrier family 7 member 11 (SLC7A11) mediates the increase in cystine uptake in rotenone-exposed (ROT) PC12 cells. (A) Western blots showing the protein levels of SLC7A11 and amino acid transporter heavy chain SLC3A2 in PC12 cells after exposure to different concentrations of ROT. (B, C) Bar graphs of the protein levels of SLC3A2 and SLC7A11 in ROT and control (CON) PC12 cells. (D, E) mRNA expression of *SLC7A11* and *SLC3A2* in ROT and control (CON) PC12 cells. (F) Fluorescence images of cystine-fluorescein isothiocyanate (FITC; green) in ROT PC12 cells treated with the SLC7A11 inhibitor sulfasalazine (SSZ). (G) Average fluorescence intensity of cystine-FITC in ROT PC12 cells treated with SSZ. (H) Fluorescence images of the cytoskeleton (red) in ROT PC12 cells treated with SSZ. (I) Cell viability of ROT PC12 cells treated with SSZ. Values are presented as the mean ± standard error of the mean (*n* ≥ 3).**P < 0.01, ***P < 0.001. BF: Bright-field; ns: not significant; SLC3A2: solute carrier family 3 member 2; SLC7A11: solute carrier family 7 member 11; SSZ: sulfasalazine.

SLC7A11 is a member of the solute carrier family and can facilitate the cellular uptake of cystine and the release of glutamate. The expression levels of SLC7A11 and its partner protein SLC3A2 were both upregulated in PC12 cells after rotenone exposure (**Figure 3A–C**). Moreover, the mRNA levels of SLC7A11 and SLC3A2 were also increased (**Figure 3D** and **E**). When sulfasalazine (SSZ), an SLC7A11 inhibitor, was used to inhibit protein activity, the cystine content was notably reduced, the cytoskeleton morphology was significantly improved (**Figure 3F–H**), and PC12 cell viability was reversed (**Figure 3I**).

### Extracellular matrix protein 1 promotes solute carrier family 7 member 11 expression in rotenone-induced PC12 cells

**Figure 4:**
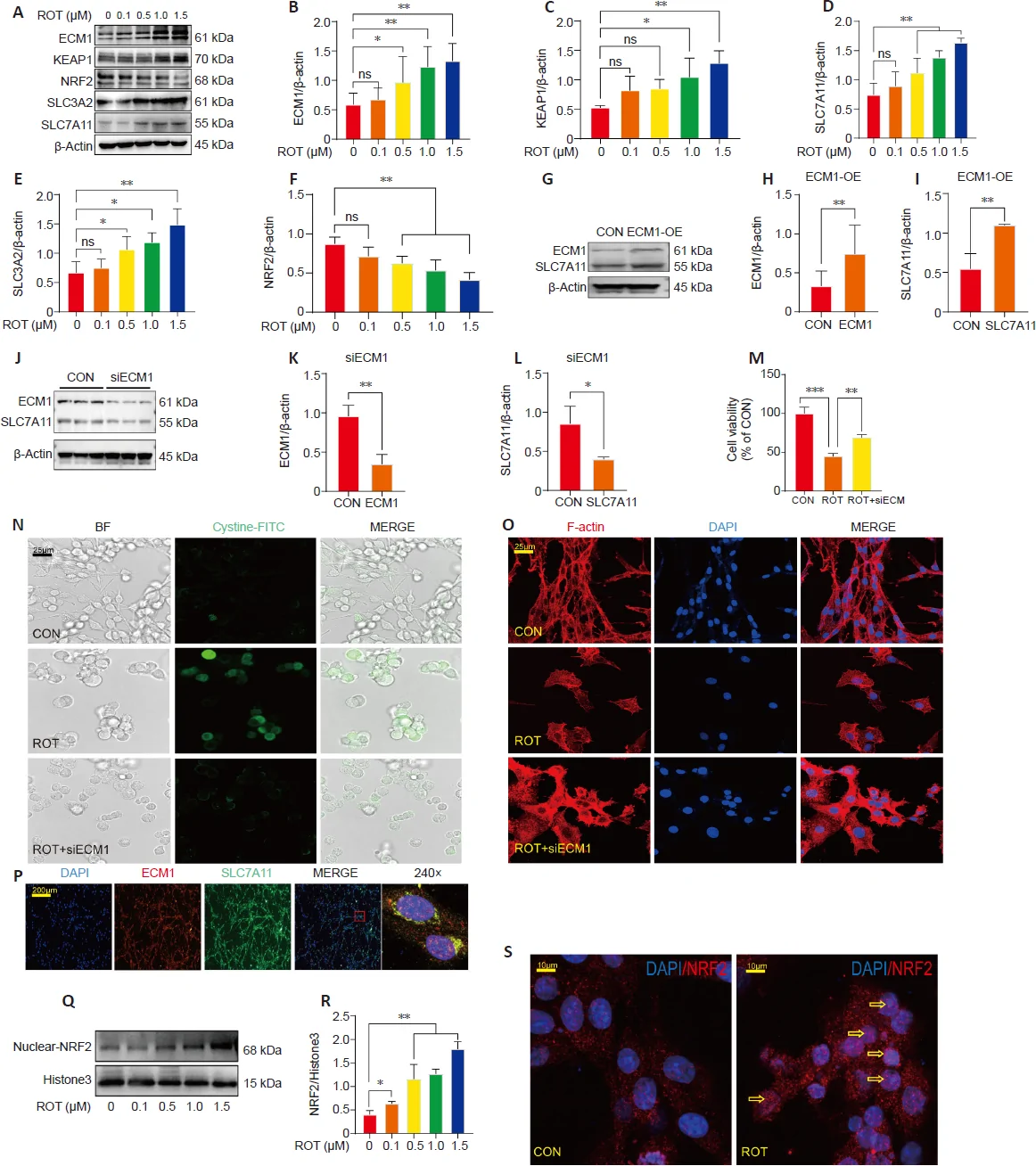
Extracellular matrix protein 1 (ECM1) promotes solute carrier family 7 member 11 (SLC7A11) expression in rotenone-exposed (ROT) PC12 cells. (A) Western blots showing the expression levels of related proteins in PC12 cells after exposure to different concentrations of ROT. (B–F) Bar graphs of the expression levels of related proteins in PC12 cells after exposure to different concentrations of ROT. (G) Protein levels of SLC7A11 and ECM1 in PC12 cells after ECM1 overexpression, and in control (CON) PC12 cells. (H, I) Bar graphs of the protein levels of SLC7A11 and ECM1 in PC12 cells after ECM1 protein overexpression. (J) Protein levels of ECM1 and SLC7A11 in PC12 cells treated with *ECM1* siRNA. (K, L) Bar graphs of the protein levels of ECM1 and SLC7A11 in PC12 cells treated with *ECM1* siRNA. (M) Cell viability of ROT PC12 cells treated with ECM1 siRNA. (N) Fluorescence images of cystine-fluorescein isothiocyanate (FITC) in ROT PC12 cells treated with *ECM1* siRNA. (O) Fluorescence images of the cytoskeleton (red) in ROT PC12 cells treated with *ECM1* siRNA. (P) Fluorescence images of ECM1 and SLC7A11 co-localization in PC12 cells. (Q) Protein levels of nuclear factor erythroid 2-related factor 2 (NRF2) in the nuclei of PC12 cells exposed to different concentrations of ROT. (R) Bar graph of the protein levels of NRF2 in the nuclei of PC12 cells exposed to different concentrations of ROT. (S) Fluorescence images of NRF2 nuclear translocation (arrows) in ROT PC12 cells. Blue is 4′,6-diamidino-2-phenylindole (DAPI), red is NRF2. Values are presented as the mean ± standard error of the mean (*n* ≥ 3). *P < 0.05, **P < 0.01, ***P < 0.001. BF: Bright-field; NRF2: nuclear factor erythroid 2-related factor 2; ns: not significant.

To explore the regulation of SLC7A11 protein, ECM1 was assayed in rotenone-exposed PC12 cells. The protein expression of ECM1 was increased in rotenone-exposed PC12 cells, which was in line with the trend of SLC7A11 protein expression. However, the ratio of NRF2 to KEAP1 was not in direct proportion to SLC7A11 expression (**Figure 4A–F**). To clarify the upstream and downstream relationships between ECM1 and SLC7A11 in rotenone-exposed PC12 cells, a lentiviral vector for ECM1 overexpression was used. The expression of SLC7A11 was upregulated with ECM1 overexpression. Furthermore, *ECM1* siRNA was used to knockdown ECM1 expression in PC12 cells. When ECM1 protein expression was knocked down, SLC7A11 protein expression was also reduced (**Figure 4G–L**). In addition, cell viability was reversed in rotenone-exposed PC12 cells with *ECM1* siRNA intervention (**Figure 4M**). Compared with the rotenone-exposed group, the cystine content was significantly decreased (**Figure 4N**), and the integrity of the cytoskeleton was restored (**Figure 4O**) in rotenone-exposed PC12 cells with *ECM1* siRNA treatment. To further clarify the regulatory role of ECM1 on SLC7A11, laser confocal microscopy was used to observe the co-localization of ECM1 and SLC7A11; there was clear co-localization of the two proteins in PC12 cells (**Figure 4P**). Additionally, NRF2 protein expression decreased and KEAP1 protein expression increased in the cytoplasm of rotenone-exposed PC12 cells, whereas NRF2 protein expression was upregulated in the nucleus. In immunofluorescence experiments, the nuclear translocation of NRF2 was observed in rotenone-exposed PC12 cells (**Figure 4Q–S**).

### The Ras-related C3 botulinum toxin substrate 1/WAVE regulatory complex/actin-related protein 2/3 pathway is activated to induce cytoskeleton collapse in rotenone-exposed PC12 cells

**Figure 5:**
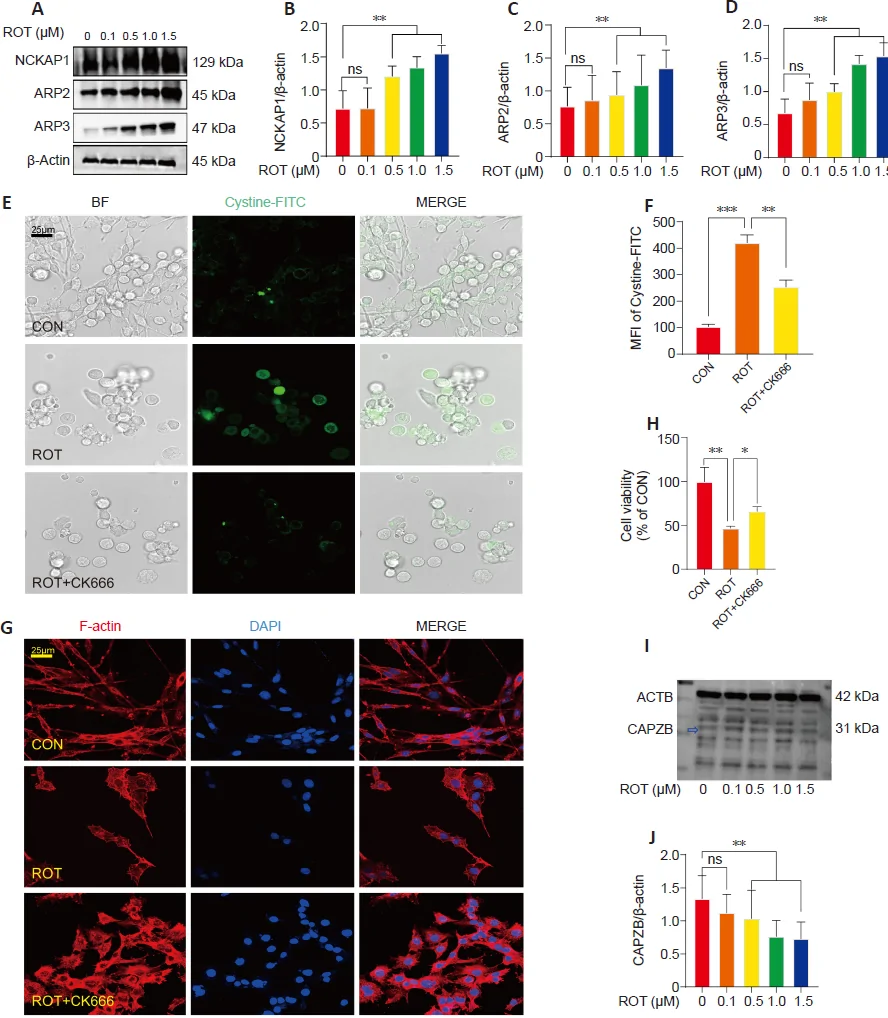
The Ras-related C3 botulinum toxin substrate 1 (RAC1)/WAVE regulatory complex (WRC)/actin-related protein (ARP)2/3 pathway is activated to induce cytoskeleton collapse in rotenone-exposed (ROT) PC12 cells. (A) Protein levels of NCK-associated protein 1 (NCKAP1), actin-related protein (ARP)2, and ARP3 in PC12 cells following exposure to various concentrations of ROT. (B–D) Bar graphs of protein levels of NCKAP1, ARP2, and ARP3 in PC12 cells exposed to different concentrations of ROT. (E) Fluorescence images of cystine-fluorescein isothiocyanate (FITC) after treatment with the ARP2-specific inhibitor CK-666 in ROT PC12 cells, and in control (CON) PC12 cells. (F) Average fluorescence intensity of cystine-FITC in ROT PC12 cells treated with CK-666. (G) Fluorescence images of the cytoskeleton in ROT PC12 cells treated with CK-666. (H) Cell viability in ROT PC12 cells treated with CK-666. (I) Western blot showing the protein expression of capping actin protein of muscle Z-line subunit beta (CAPZB) in ROT PC12 cells. (J) Bar graph of the protein levels of CAPZB in PC12 cells exposed to different concentrations of ROT. Values are presented as the mean ± standard error of the mean (*n* ≥ 3). *P < 0.05, **P < 0.01, ***P < 0.001. BF: Bright-field.

The expression levels of the key protein NCKAP1 in the WAVE regulatory complex (WRC) complex and the actin-polymerizing proteins actin-related protein (ARP2/3) were all upregulated (**Figure 5A–D**) in rotenone-exposed PC12 cells. Subsequently, the ARP2-specific inhibitor CK-666 was used in rotenone-exposed PC12 cells. The cystine content inside the cells decreased (**Figure 5E** and **F**) in rotenone-exposed PC12 cells treated with CK-666 compared with the rotenone-exposed group. Additionally, the morphology of the cytoskeleton was also restored to some degree (**Figure 5G**) in rotenone-exposed PC12 cells treated with CK-666, and the viability of PC12 cells was notably reversed (**Figure 5H**) with CK-666 treatment compared with the control group. Furthermore, the protein expression of CAPZB, which is the tail-end structure of F-actin, was decreased with increasing rotenone concentrations (**Figure 5I** and **J**).

### Abnormal disulfide bond formation is promoted in rotenone-exposed PC12 cells

**Figure 6:**
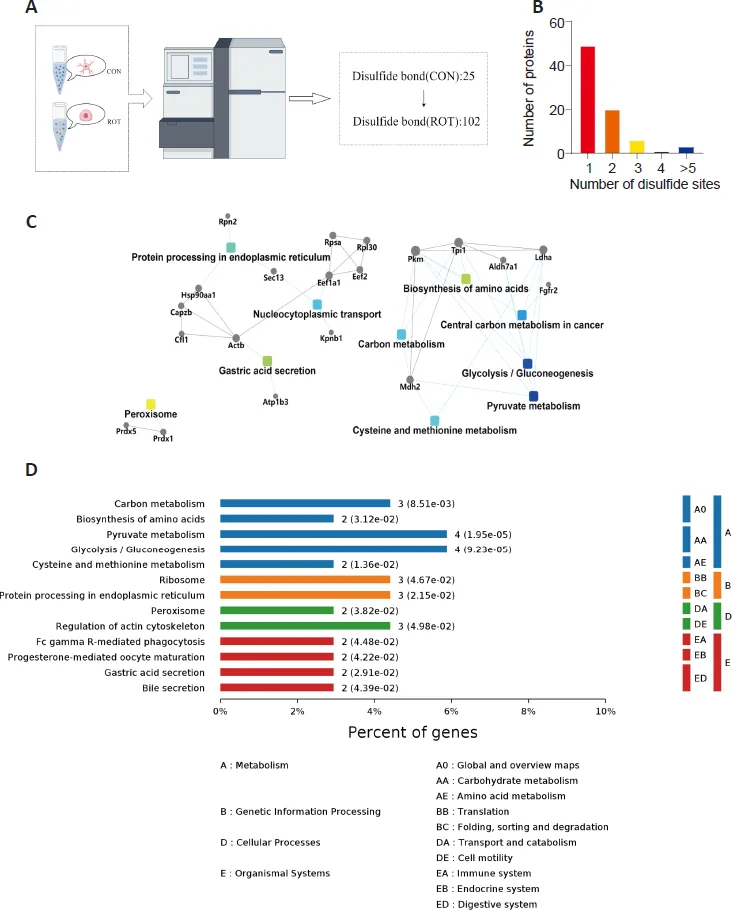
Formation of abnormal disulfide bonds was promoted in rotenone-exposed PC12 cells. (A) The number of disulfide bonds was calculated in rotenone-exposed PC12 cells. (B) The number of proteins with different numbers of abnormal disulfide bonds in rotenone-exposed PC12 cells. (C) Diagram of the protein relationships participating in abnormal disulfide bond formation in rotenone-exposed PC12 cells. (D) Gene Ontology (GO) enrichment analysis from the Gene Ontology Resource.

After conducting disulfide bond omics detection and analyzing the formation of the disulfide bonds in rotenone-exposed PC12 cells, it was noted that the number of disulfide bonds in rotenone-exposed PC12 cells increased sharply, from 25 in untreated cells to 102 in rotenone-exposed cells (**Figure 6A** and **B**). Furthermore, the interactions among the involved proteins were analyzed using STRING software. Multiple cytoskeletal proteins were involved in the formation of abnormal disulfide bonds, including CAPZB, anillin, and cofilin-1 (**Figure 6C**). Moreover, KEGG enrichment analysis demonstrated that multiple cytoskeleton-related pathways were activated in rotenone-exposed PC12 cells (**Figure 6D**).

### Intervention of disulfide stress improves rotenone-exposed PC12 cell function

**Figure 7:**
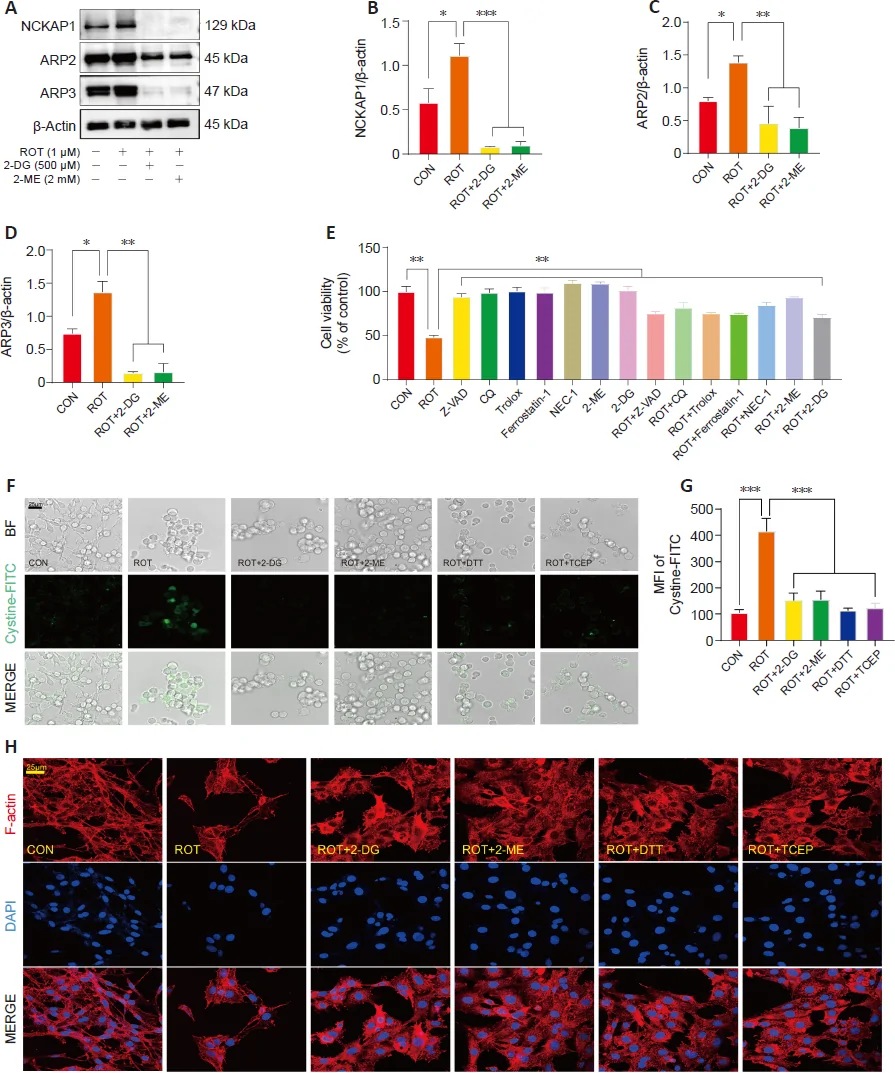
Interventions targeting disulfide stress improved rotenone-exposed (ROT) PC12 cell function. (A) Protein levels of actin-related protein (ARP)2, ARP3, and NCK-associated protein 1 (NCKAP1) in control (CON) and ROT PC12 cells treated with 2-mercaptoethanol (2-ME) or 2-deoxy-D-glucose (2-DG). (B–D) Bar graphs of the protein levels of NCKAP1, ARP2, and ARP3 in ROT PC12 cells treated with various death inhibitors and disulfide bond reducing agents. (E) Cell viability of ROT PC12 cells treated with various death inhibitors. (F) Fluorescence images of cystine-fluorescein isothiocyanate (FITC; green) in ROT PC12 cells treated with 2-ME, dithiothreitol (DTT), Tris(2-carboxyethyl) phosphine (TCEP), or 2-DG. (G) Average fluorescence intensity of cystine-FITC in ROT PC12 cells treated with 2-ME, DTT, TCEP, or 2-DG. (H) Fluorescence images of the cytoskeleton (red) in ROT PC12 cells treated with 2-ME, DTT, TCEP, or 2-DG. Values are presented as the mean ± standard error of the mean (*n* ≥ 3). *P < 0.05, **P < 0.01, ***P < 0.001. BF: Bright-field; CAPZB: actin-capping protein beta subunit; NCKAP1: NCK-associated protein 1.

The cystine content was significantly increased and cystine accumulated in the cytoplasm in rotenone-exposed PC12 cells, leading to intracellular disulfide stress. Therefore, 2-DG (a glucose analog) and 2-ME (a thiol-reducing agent) were used to reduce cystine accumulation in PC12 cells. Cell viability was significantly restored in rotenone-exposed PC12 cells treated with 2-DG or 2-ME. Additionally, NCKAP1 and ARP2/3 expression was also significantly inhibited (**Figure 7A–D**) in rotenone-exposed PC12 cells treated with the 2-ME or 2-DG. To confirm the toxic effect of rotenone-induced intracellular disulfide stress in PC12 cells, the cells were treated with various death inhibitors and disulfide bond reducing agents. All treatments were able to significantly, but not completely, reverse the viability of rotenone-exposed PC12 cells (**Figure 7E**). Moreover, in rotenone-exposed PC12 cells treated with 2-DG, 2-ME, DTT, or TCEP, the immunofluorescence intensity of cystine was significantly reduced (**Figure 7F** and **G**) and the collapse of the cytoskeleton was significantly improved (**Figure 7H**). These results further indicate that disulfide stress, caused by the rotenone-induced influx of cystine, plays an important role in the development of cell death and the collapse of the cytoskeleton, which has been termed disulfidptosis.

### Disulfidptosis is induced in rotenone-exposed rats

**Figure 8:**
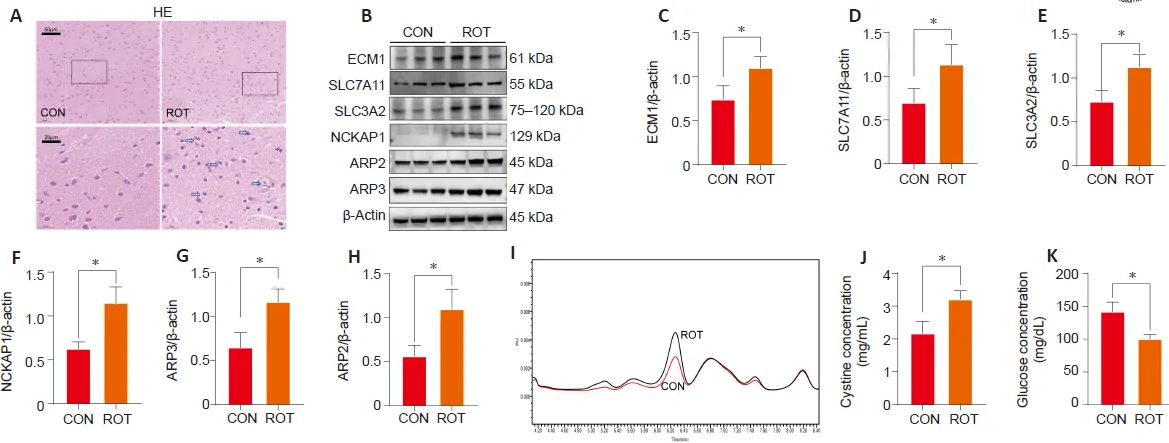
Disulfidptosis was induced in rotenone-exposed (ROT) rats. (A) Hematoxylin and eosin (HE) staining in the striatum of rats in the ROT and control (CON) groups. The morphology of neurons (arrows) in the striatum was destroyed, and many vacuolated nuclei were observed in ROT rats. (B) Western blots showing the protein levels of ECM1, SLC7A11, amino acid transporter heavy chain SLC3A2, NCKAP1, ARP2, and ARP3 in the striatum of ROT and CON rats. (C–H) Bar graphs of the protein levels of ECM1, SLC7A11, SLC3A2, NCKAP1, ARP2, and ARP3 in the striatum of ROT and CON rats. (I) Peak chart of cystine, detected using high-performance liquid chromatography, in the striatum of ROT and CON rats. (J) Cystine content in the striatum of ROT and CON rats. (K) Glucose content in the striatum of ROT and CON rats. Values are presented as the mean ± standard error of the mean (*n* = 6). *P < 0.05. ARP2: Actin-related protein 2; ARP3: actin-related protein 3; ECM1: extracellular matrix protein 1; NCKAP1: NCK-associated protein 1; NRF2: nuclear factor erythroid 2-related factor 2; SLC7A11: solute carrier family 7 member 11.

A PD model was established by exposing rats to rotenone; this model, which has been previously validated using neurobehavioral and pathological studies (Xiao et al., 2023; Yamamoto et al., 2024). Hematoxylin and eosin staining revealed damaged striatal neurons in the rotenone exposure group; specifically, the nuclei of neuronal cells were evenly stained dark purple, and empty nuclei were observed in some cells (**Figure 8A**). Moreover, compared with the control group, the striatal protein expression levels of ECM1, SLC7A11, SLC3A2, NCKAP1, ARP2, and ARP3 were significantly higher in the rotenone exposure group (**Figure 8B–H**). Furthermore, the striatal cystine content was higher in the rotenone exposure group than in the control group (**Figure 8I** and **J**), whereas the striatal glucose content was decreased (**Figure 8K**).

## Discussion

PD is a neurodegenerative disorder with a highly complex etiology and pathogenesis (Chernivec et al., 2018; Assogna et al., 2020; Asanuma and Miyazaki, 2021; Bidesi et al., 2021; Cong et al., 2022). Rotenone is a biogenic pesticide that can inhibit the activity of mitochondrial complex I. Numerous studies have reported that rotenone exposure can heighten the risk of PD (Eira et al., 2016; Dodiya et al., 2020; Downs et al., 2022). Our research team has focused on the mechanisms by which PD-like symptoms are induced by rotenone. In our experiments, the rotenone-induced collapse of the cytoskeleton was observed clearly in dopaminergic neurons. This finding led to our strong interest in cytoskeletal changes of rotenone-exposed cells.

In the present study, cell morphology and viability was significantly altered in rotenone-exposed PC12 cells. The morphological changes were manifested as the shortening of processes and the transformation from a long spindle shape into a round or irregular shape. These morphological alterations in PC12 cells are not merely a superficial visual change and may involve a series of complex physiological and pathological processes. We therefore further investigated the effects of rotenone on the cytoskeleton of PC12 cells. The overall cytoskeleton was severely damaged in rotenone-exposed PC12 cells, presenting an irregular state. Microtubules and microfilaments are important structures of the cytoskeleton. Under normal circumstances, microtubules play a crucial role in maintaining cell morphology and participating in material transportation (Sen et al., 2022; Yang et al., 2022; Sisario et al., 2024). By contrast, microfilaments mainly participate in cell movement, contraction, and other functions (Mi et al., 2015; Liu et al., 2023; Roy et al., 2023). In PC12 cells, rotenone exposure may lead to microtubule depolymerization and disrupt the polymerization balance of tubulin, subsequently resulting in the instability of the microtubule structure of the cytoskeleton. This destruction of the microtubule structure of the cytoskeleton then leads to changes in PC12 cell morphology, such as cell rounding and the retraction of processes. Simultaneously, rotenone may cause a disorder of microfilaments in PC12 cells, decrease their adhesion ability, and weaken motility. Cytoskeleton abnormalities may therefore play an important role in rotenone-induced PC12 cell damage.

Disulfidptosis is a newly identified form of cell death that is induced by disulfide stress arising from excessive cystine accumulation in cells. This type of cell death was initially reported in 2023 (Liu et al., 2023). Disulfidptosis can give rise to abnormal disulfide bonds between actin cytoskeleton proteins, which results in cytoskeleton collapse and, ultimately, cell death (Hadian and Stockwell, 2023; Hu et al., 2023; Liu et al., 2023). In the present study, through proteomic analysis, a number of important disulfidptosis-related proteins were identified as significantly different between PC12 cells exposed to rotenone and the control group. This result was consistent with the observed changes in the analysis of the GEO PD database. By integrating and analyzing PD-related data from the GEO database, known disulfidptosis-related proteins were revealed to exhibit differences between PD patients and control individuals. Furthermore, with the analysis of disulfide bond omics, the number of abnormal disulfide bonds was clearly increased in rotenone-treated PC12 cells compared with the control group.

Cystine is a sulfur-containing amino acid that is formed by the combination of two cysteine molecules via a disulfide bond; it holds a unique position in cellular physiological processes. It is widely involved in various biochemical reactions, such as protein synthesis and folding, as well as antioxidant reactions (Koppula et al., 2018; Asanuma and Miyazaki, 2021; Han et al., 2024). HPLC and cystine-FITC live cell staining experiments revealed that the cystine content was significantly increased in rotenone-exposed PC12 cells compared with the control group. From a molecular perspective, this increase in cystine may alter the redox environment inside the cell, and this environmental change may further affect the structure and function of a series of cytoskeleton-related proteins.

In further *in vitro* and *in vivo* experiments, rotenone exposure was demonstrated to significantly increase SLC7A11 and SLC3A2 protein expression. SLC7A11 is a cystine transporter whose function is to transport cystine from outside the cell to inside the cell. The cystine that enters the cell is further synthesized into GSH and inhibits the occurrence of cellular oxidative stress. When the SLC7A11 protein inhibitor SSZ was used to treat rotenone-exposed PC12 cells, the cystine content was significantly reduced, and PC12 cell activity was effectively improved. This experimental result further indicates that the cystine transporter SLC7A11 is involved in cystine accumulation in rotenone-exposed PC12 cells. Excessively accumulated cystine might lead to increased intracellular disulfide stress, thereby reducing the active state of cells. After the SSZ treatment of rotenone-exposed PC12 cells, the cell morphology and cytoskeleton alterations were reversed; cells changed from a round or irregular shape into a long spindle shape, and the cytoskeleton arrangement became more regular. This further indicates that the cystine transporter SLC7A11 plays an important role in rotenone-exposed PC12 cells. By excessively exporting extracellular cystine, it induced intracellular disulfide stress, which in turn led to toxic effects on the cytoskeleton.

It has been reported that the process of reducing cystine to cysteine within cells demands a large amount of NADPH (Asanuma and Miyazaki, 2021). NADPH mainly originates through the glucose/pentose phosphate metabolic pathway. Glucose content and metabolism were therefore detected in rotenone-exposed PC12 cells. The glucose content exhibited a significant decrease in rotenone-exposed PC12 cells. This reduction in glucose content might further influence the production of NADPH in PC12 cells. Under normal circumstances, a large quantity of NADPH is required during the process of reducing cystine to cysteine (Asanuma and Miyazaki, 2021). When the NADPH pool is depleted in rotenone-exposed PC12 cells, the conversion of cystine to cysteine is inhibited, leading to an increased cystine content in cells (Liu et al., 2020). Excessive cystine then induces disulfide stress in cells, which results in an abnormal cytoskeleton and triggers disulfidptosis (Liu et al., 2023). In the present study, the glucose analog 2-DG was used to counter the toxic effects of rotenone exposure in PC12 cells. Cell viability was significantly increased in rotenone-exposed PC12 cells treated with 2-DG compared with rotenone-exposed PC12 cells. Moreover, the cell morphology and cytoskeleton alterations were reversed, changing from a round or irregular shape to a long spindle shape in the rotenone-exposed PC12 cells treated with 2-DG. These changes might be attributed to the improvement by 2-DG of NADPH content and the promotion of cystine to cysteine conversion in rotenone-exposed PC12 cells.

In summary, the expression of SLC7A11 protein was upregulated in rotenone-exposed PC12 cells, which resulted in the increased transport of cystine from the outside of PC12 cells to the inside of PC12 cells. Furthermore, rotenone exposure affected glucose metabolism and led to decreased cellular NADPH content, thereby inhibiting the conversion of intracellular cystine to cysteine. The combined effect of these two factors led to a sharp increase in cystine content in PC12 cells. Finally, the excessive accumulation of cystine promoted disulfide stress in the rotenone-exposed PC12 cells.

In the current study, expression of the extracellular matrix protein ECM1 in PC12 cells increased with increasing rotenone concentrations. ECM1 overexpression and siRNA treatment revealed that changes in SLC7A11 protein expression were closely associated with changes in ECM1 protein expression. Additionally, immunofluorescence experiments demonstrated that ECM1 and SLC7A11 were co-localized in PC12 cells. Similarly, it has been reported that ECM1 can regulate SLC7A11 expression (Fu et al., 2024). Moreover, the inhibition of ECM1 activity effectively restrained SLC7A11 activity. Rotenone might therefore affect SLC7A11 expression levels by influencing the role of ECM1, thus triggering a series of cascade reactions. In the present study, we observed an increase in nuclear NRF2 expression after rotenone exposure. Notably, the targeted regulation of SLC7A11 by nuclear NRF2 has been confirmed in a previous study (Liu et al., 2022). In the case of rotenone, the observed increase in NRF2 may therefore represent a compensatory response to counteract the oxidative damage induced by complex I inhibition. This phenomenon may be related to the drug dosage and the treatment duration.

Many previous studies have reported that SLC7A11 plays an important role in maintaining intracellular GSH levels and protecting cells from oxidative stress-induced cell death (Koppula et al., 2018; Li et al., 2021; Huang et al., 2022; Han et al., 2024). However, in recent years, an increasing number of reports have noted that under glucose starvation conditions, high SLC7A11 expression promotes cell death via disulfidptosis (Liu et al., 2023; Ma et al., 2023; Wang et al., 2023b; Song et al., 2025). Together, these results indicate that disulfidptosis may be involved in the damage of dopaminergic neurons exposed to rotenone.

Rotenone exposure leads to disulfide stress within cells, activates the Ras-related C3 botulinum toxin substrate 1 (RAC1)/WRC/ARP2/3 pathway, and upregulates the expression of ARP2/3 (Yang et al., 2022; van Eeuwen et al., 2023; Sisario et al., 2024). ARP2/3 is an F-actin polymerization protein that is normally attached to the side of F-actin. When its expression is upregulated, the branched actin of F-actin captures monomeric G-actin and polymerizes into a branched network of F-actin, thus providing a target for cellular actin to participate in the formation of abnormal disulfide bonds (van Eeuwen et al., 2023). Our experiments revealed that the expression of both NCKAP1 and ARP2/3, which are related to the RAC1/WRC/ARP2/3 pathway, was significantly upregulated in rotenone-exposed PC12 cells. The accumulation of intracellular cystine might further activate the RAC1/WRC/ARP2/3 pathway, thereby creating favorable conditions for an increase in the F-actin branch network. CK-666 is an inhibitor of ARP2 (Chakrabarti et al., 2022; Oliveira et al., 2023). In the current study, cell viability was significantly improved in rotenone-exposed PC12 cells treated with CK-666 compared with cells that underwent rotenone exposure only. Moreover, CK-666 treatment also reversed rotenone-induced changes in the cell morphology and cytoskeleton of PC12 cells, which changed from a round or irregular shape to a long spindle shape. CK-666 might therefore inhibit ARP2, thus decreasing F-actin accumulation and reducing the target of abnormal disulfide bond formation between cytoskeleton proteins. These findings prompted us to hypothesize that the ARP2/3 complex might act as a terminal target for regulating the dynamic balance of F-actin. ARP2/3 is an actin polymerization protein that plays a key nucleation role in actin filament assembly and can initiate the formation of new actin filaments. It regulates cell morphology and movement and promotes the polymerization of actin filaments at the front edge of cells to form protrusion structures during cell migration. The activated pathway promotes F-actin polymerization (Romani et al., 2022; Carman et al., 2023; Liu et al., 2023; van Eeuwen et al., 2023). As an important part of the cytoskeleton, the polymerization process of F-actin provides sufficient targets for the formation of abnormal disulfide bonds, ultimately leading to disulfidptosis. In the current study, rotenone exposure led to significantly reduced CAPZB expression. The upregulated expression of the actin polymerization protein ARP2 and the reduced expression of the capping protein CAPZB, which regulates the “bottom line” of F-actin polymerization, may have allowed the F-actin branch network structure to polymerize better and provided sufficient targets for the formation of abnormal disulfide bonds (Mi et al., 2015; Funk et al., 2021). Unexpectedly, however, the cystine content of cells was also reduced after CK-666 treatment. This finding may be associated with the establishment of a negative feedback mechanism that resulted from the dynamic equilibrium of the intracellular actin network.

The expression of NCKAP1 and ARP2/3 proteins was significantly improved with 2-DG treatment and the NADPH content was increased in rotenone-exposed PC12 cells. Moreover, the expression of NCKAP1 and ARP2/3 proteins in rotenone-exposed PC12 cells was also significantly improved with 2-ME, DTT, or TCEP treatment. These results further indicate that disulfide stress-induced abnormal cytoskeleton protein expression plays an important role in rotenone-induced dopaminergic neuron degeneration and damage. Consequently, the cytoskeleton likely plays a vital role during the disulfidptosis process, and its stability and function are crucial for maintaining the normal physiological activities of cells.

The detection and analysis of disulfide proteomics were conducted in rotenone-exposed PC12 cells. The number of disulfide bonds increased significantly from 25 pairs in untreated PC12 cells to 102 pairs in rotenone-exposed PC12 cells. Further analysis indicated that the proteins involved in the formation of these abnormal disulfide bonds included many cytoskeleton-related proteins. Rotenone upregulated SLC7A11 protein expression in PC12 cells and subsequently led to cystine accumulation. It might be that the increase in cystine led to the formation of abnormal disulfide bonds of cytoskeleton proteins, which manifested as the collapse of the PC12 cytoskeleton. However, a significant increase in cystine may also lead to complex interactions with other metabolites in cells (Koppula et al., 2018). For example, it might affect the metabolic balance of other sulfur-containing compounds, such as the synthesis and decomposition of antioxidant substances (e.g., GSH), or it may react with some antioxidant substances, thereby indirectly affecting the cytoskeleton stability (Zhong et al., 2023).

In the current experiments, drugs that reduce disulfide bonds (e.g., 2-ME, TCEP, and DTT) were administered to rotenone-exposed PC12 cells. With these treatments, PC12 cell activity increased and the morphology of the cytoskeleton was restored, indicating that disulfide accumulation is a key factor leading to alterations in the morphology of the cytoskeleton in PC12 cells. These findings further suggest that cellular disulfide stress caused by cystine accumulation is the main reason for the collapse of the cytoskeleton in rotenone-exposed PC12 cells.

The identification of disulfidptosis as a novel cell death mechanism has opened new avenues for therapeutic interventions in neurodegenerative diseases, including PD. On the basis of our findings, several potential strategies may be explored to target disulfidptosis and its underlying pathways. First, the targeting of key enzymes involved in disulfide bond formation or reduction, such as thioredoxin reductase and protein disulfide isomerase, represents a promising approach. Small-molecule inhibitors or activators of these enzymes might be developed to restore disulfide homeostasis and prevent cytoskeletal protein aggregation. Additionally, the stabilization of cytoskeletal proteins through peptide-based therapies or small molecules may offer a direct means of preventing disulfidptosis (Liu et al., 2020). Second, the enhancement of cellular antioxidant defenses through gene therapy might provide a broad protective effect against disulfide stress (Romani et al., 2022). Although these strategies hold great potential, further research is needed to validate their efficacy and safety in preclinical and clinical settings. The development of targeted therapies for disulfidptosis may ultimately lead to novel treatments for neurodegenerative diseases, thus addressing an unmet medical need (Vallerga et al., 2020).

To date, disulfidptosis has been predominantly explored within tumor cell models, with no previous investigations in neurodegenerative models. In the present study, rotenone exposure led to an upregulation of SLC7A11 protein expression and induced a cystine influx, which led to intracellular disulfide stress and ultimately resulted in disulfidptosis. Although the research was constructive, only *in vitro* experiments were conducted in the current study. The next step will be to further validate the relevant conclusions through *in vivo* experiments.

The present study has some limitations. First, human tissue samples and blood specimens are required to validate the expression of SLC7A11—a key gene linked to disulfidptosis—and alterations in cystine levels and glucose concentrations in patients with PD. Second, in-depth investigations into the effects of rotenone on the pentose phosphate pathway are required to further elucidate the mechanisms underlying the rotenone-mediated inhibition of NADPH production.

In conclusion, our findings allow a deeper understanding of the pathogenesis of PD. One of the core pathological characteristics of PD is the progressive degeneration and death of dopaminergic neurons. The excessive accumulation of cystine can lead to disulfidptosis in rotenone-exposed dopaminergic neurons; this finding may offer a new direction for etiological research in PD. The cell death mechanism caused by cystine accumulation might play an important role in PD and related diseases, which provides a new perspective for research into these diseases. Adjuvant treatment of PD may be achieved by developing drugs that target the cystine metabolic pathway or regulate cystine-related intracellular signaling pathways.

## Data Availability

*No additional data are available.*
